# Prevalence and Factors Associated With Post-Cholecystectomy Syndrome in Saudi Arabia

**DOI:** 10.7759/cureus.32827

**Published:** 2022-12-22

**Authors:** Mohammad Eid M Mahfouz, Abdulmohsen Dubayyan M Altowairqi, Hussam Y Alghamdi, Mazen Saeed Z Alzahrani, Asim K Alqurashi, Talal H Alhuraity, Amr S Alqurashi

**Affiliations:** 1 Surgical, Taif University, Taif, SAU; 2 College of Medicine, Taif University, Taif, SAU

**Keywords:** saudi arabia, gallstones, cholecystectomy, post-cholecystectomy syndrome, prevalence

## Abstract

Objectives: Post-cholecystectomy syndrome (PCS) refers to the continuation or recurrence of biliary colic and any other gastrointestinal symptoms present prior to cholecystectomy. Given that PCS is rare and underestimated in Saudi Arabia, this study aimed to determine the prevalence of PCS among Saudis.

Methods: This cross-sectional study included randomly gathered subjects who self-administered the validated European Organization for Research and Treatment of Cancer QLQ-C30 questionnaire from September 6 to October 7, 2022.

Results: A total of 518 participants who underwent cholecystectomies were included; most were female (73.6%), 153 (29.5%) were 19 to 25 years old, and the vast majority were Saudi Arabian (91.1%). The average preoperative weight was 71 ± 20.7 kg (range 20 to 258), and the mean current weight was 69 ± 19.4 kg (range 30 to 257). About 137 (26.4%) had a chronic disease, most frequently hypertension (37.2%). In the past week, most participants experienced some trouble doing strenuous activities (42.9%), slight pain (37.6%), fatigue (32.2%), insomnia (37.5%), and weakness (38.2%). There were no significant associations between age, nationality, or residence with the QLQ-C30 score (P-value = 0.152, 0.262, 0.071, respectively). A significant association was observed between gender and QLQ-C30 score (P-value < 0.001), with females scoring higher than their male counterparts. Females also reported having a higher quality of life than males.

Conclusions: The prevalence of PCS was higher than reported elsewhere.

## Introduction

Gallstones and other gallbladder problems are commonly treated with cholecystectomy [1]. Laparoscopic cholecystectomy is a minimally invasive surgical procedure to remove the gallbladder [2]. Currently, laparoscopic cholecystectomy is indicated for treating cholecystitis (acute/chronic), symptomatic choledocholithiasis, biliary dyskinesia, biliary pancreatitis, and masses/polyps of the gallbladder [3]. The first cholecystectomy was performed in 1882 by Carl Langebuch (1846-1901). Prof. Dr. Erich Mühe of Germany performed the world's first laparoscopic cholecystectomy in 1985, 103 years later [4].

Gallstone disease continues to be one of the most common digestive disorders in the world. The rate of gallstone formation increases with age. In recent years, ultrasonographic data on the incidence of gallstones have been reported. The reported incidence of gallstones in the United States is about 10%-15%, with millions of new cases diagnosed each year. Although the true incidence of gallstones in Saudi Arabia is unknown.

Since its successful implementation in 1987, laparoscopic cholecystectomy (LC) has rapidly become the standard treatment for symptomatic gallstone disease. It is already well-established in Saudi Arabia [5]. Gallstones are classified into two categories based on their chemical composition and macroscopic appearance (cholesterol and pigment gallstones). The primary cause of cholesterol gallstone formation is hepatic hypersecretion. As a result, gallbladder hypomotility and rapid phase transitions occur. Intestinal factors, such as increased cholesterol absorption and decreased bile salt absorption, contribute to cholesterol gallstone formation. Pigmented gallstones are associated with abnormal bilirubin metabolism, patients with black or brown pigmented stones have high levels of unconjugated bilirubin in their bile [6].

In general, the diagnosis, clinical symptoms, and management of gallstones are the same for cholesterol and pigment stones but mostly depend on the location of the stone. Patients with gallbladder stones may experience biliary colic, which is described as episodic attacks of severe pain in the right upper quadrant or epigastrium lasting at least 20-30 minutes, with pain radiating to the right back or shoulder, which improves with analgesics [6].

Abdominal ultrasonography and endoscopic retrograde cholangiography are the preferred modalities for diagnosis [6]. Cholecystectomy is indicated in cases of gallbladder trauma and gallbladder cancer, including porcelain gallbladders, gallstone complications, acute cholecystitis, and in most patients with symptomatic cholelithiasis (chronic cholecystitis), especially those with non-functioning gallbladders. Some patients with chronic cholecystitis are candidates for gallstone lithotripsy, and an even smaller number for gallstone dissolution [7].

Post-cholecystectomy syndrome (PCS) refers to the continuation or recurrence of upper abdominal pain (primarily the right upper quadrant) and several gastrointestinal symptoms comparable to those seen before cholecystectomy. This condition might be the persistence of symptoms produced by gallbladder pathology or the appearance of new symptoms that are usually linked to the gallbladder. For the first time in 1947, Womack and Crider defined post-cholecystectomy syndrome as "the presence of symptoms after cholecystectomy” [8]. Symptoms attributed to the gallbladder can remain or return for months or years after the cholecystectomy. The most prevalent symptoms are abdominal pain, nausea, vomiting, dyspepsia, flatulence, loose stools or fecal urgency, and bloating, which usually occur before cholecystectomy; the persistence of Biliary colic is prevalent. Severe abdominal pain, jaundice, fever, and chills are uncommon; however, their occurrence indicates a higher possibility of a particular, curable cause than those with non-specific, dyspeptic, or mild symptoms [9].

PCS affects at least 15% of people, and symptoms might appear for anywhere from days to years [10]. There are two major risk factors for PCS recognized by modern medicine: biliary tract factors (for example, residual choledocholithiasis, neo-deposited stones in the common bile duct, excessive residual cystic duct, Oddi sphincter dysfunction) and non-biliary tract factors (for example, acute or chronic gastritis, peptic ulcer, malignant tumors in the gastrointestinal tract) [11]. There is a persistence or recurrence of symptoms in 20%-40% of patients [12]. Very rarely, these patients may present with abdominal pain, jaundice, or dyspepsia early after surgery. These symptoms may be due to bile duct complications, especially after gallbladder removal using laparoscopic techniques. These complications include biliary tract injury, bile ducts, fistulas, and bile duct stones retention. The long-term effects are recurrent bile duct stones and bile duct stenosis [13].

Any patient with PCS requires symptomatic treatment and a thorough workup to determine the exact cause of the symptoms; liver functions and abdominal ultrasounds are usually the first tests requested, with further investigations customized accordingly [14]. Computed tomography (CT) scan, endoscopic retrograde cholangiopancreatography (ERCP), and magnetic resonance cholangiopancreatography (MRCP) are all imaging techniques used to diagnose post-cholecystectomy syndrome [15]. The typical imaging approach to PCS involves ultrasonography and CT, with direct cholangiography serving as the gold standard. For the examination of the biliary tract, magnetic resonance cholangiopancreatography (MRCP) provides a non-invasive and reliable alternative to direct cholangiography. This has increased the use of MRCP in patients with suspected PCS, even though its role in patient care has only been briefly evaluated [16]. The approaches for managing post-cholecystectomy syndrome center on treating the underlying cause [15]. PCS treatment involves medication, sphincterotomy, biliary stenting, percutaneous drainage of bilomas, and surgical revision for severe strictures [16].

## Materials and methods

Study design

A cross-sectional study was designed to assess the prevalence of PCS among the communities of Saudi Arabia. The minimum sample size required was 385 subjects representative of the Saudi population (32 million) with a confidence level (CL) of 95% and a confidence interval (CI) of 5%. Only completed surveys were included.

Data collection

A sample of 518 individuals was collected randomly using a self-administered validated Arabic version of the European Organization for Research and Treatment of Cancer (EORTC) QLQ-C30 questionnaire on the website and trained medical students through interviewing the general population from September 6 to October 7, 2022. The socio-demographic data were assessed for all participants. In addition to the EORTC QLQ-C30 questionnaire to determine the prevalence of post-cholecystectomy syndrome (PCS) in Saudi Arabia.

EORTC QLQ-C30

The EORTC Core Quality of Life questionnaire (EORTC QLQ-C30) is designed to measure cancer patients' physical, psychological and social functions. The questionnaire is composed of multi-item scales and single items. It has been translated and validated into over 100 languages and is used each year in more than 5,000 studies worldwide.

Statistical analysis

All data were recorded in a pre-designed and validated excel sheet. Data were represented in terms of frequencies and valid percentages for categorical variables. Data were analyzed using IBM Corp. Released 2015. IBM SPSS Statistics for Windows, Version 23.0. Armonk, NY: IBM Corp. to perform all statistical calculations.

Ethical considerations

Ethics approval was obtained from the Research and Studies Department at the Directorate of Health Affairs in Taif (Approval number: 685). Participant identity was kept confidential.

## Results

A total of 518 participants who underwent cholecystectomy were enrolled in the current study; most were females (73.6%), while 137 (26.4%) were males. Regarding age groups, 19 (3.7%) of the participants were aged less than 18 years, 153 (29.5%) were 19 to 25 years old, 137 (26.4%) were 26 to 35 years old, 115 (22.2%) were 36 to 45 years old, 62 (12%) were 46 to 55 years old, and 32 (6.2%) were aged 55 years old. The vast majority were of Saudi Arabian nationality (91.1%), and 46 (8.9%) were of non-Saudi nationality. More than one-third of the participants (36.9%) were residents of Makkah, 64 (12.4%) were from Riyadh, and 56 (10.8%) were from Madinah. The average preoperative weight of the participants was 71 ± 20.7 kg (range 20 to 258), and 69 ± 19.4 kg (range 30 to 257) was their present average weight. Most of the participants were in the healthy weight range both preoperatively (36.7%) and currently (37.8%). About 137 (26.4%) had a history of chronic diseases (Table 1).

**Table 1:** Socio-demographic characteristics of the respondents (n=518)

Variable	Category	Frequency	Percent
Age (years)	< 18	19	3.7%
	19-25	153	29.5%
	26-35	137	26.4%
	36-45	115	22.2%
	46-55	62	12%
	> 55	32	6.2%
Gender	Male	137	26.4%
	Female	381	73.6%
Nationality	Saudi	472	91.1%
	Non-Saudi	46	8.9%
Residence	Al-Baha	36	6.9%
	Al-Jouf	7	1.4%
	Northern borders	6	1.2%
	Riyadh	64	12.4%
	Eastern Region	32	6.2%
	Al-Qassim	16	3.1%
	Madinah	56	10.8%
	Tabuk	20	3.9%
	Jizan	27	5.2%
	Hail	40	7.7%
	Aseer	19	3.7%
	Makkah	191	36.9%
	Najran	4	0.8%
Preoperative Body Mass Index (BMI)	Underweight	32	6.2%
	Healthy weight	190	36.7%
	Overweight	147	28.4%
	Obese	149	28.8%
Current Body Mass Index (BMI)	Underweight	35	6.8%
	Healthy weight	196	37.8%
	Overweight	150	29%
	Obese	137	26.4%
History of chronic disease	Yes	137	26.4%
	No	381	73.6%

**Figure 1:**
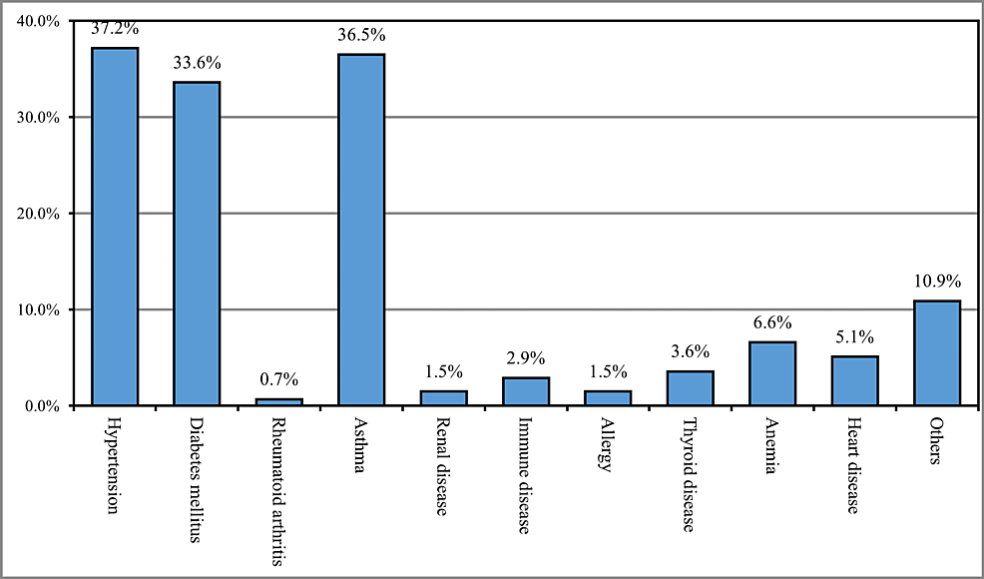
History of chronic disease (n=137)

The most reported chronic disease among the study participants was hypertension (37.2%), followed by asthma (36.5%), diabetes mellitus (33.6%), anemia (6.6%), and heart diseases (5.1%), while 10.9% had other chronic diseases (Figure 1).

To determine the prevalence of post-cholecystectomy syndrome (PCS), the EORTC QLQ-C30 questionnaire was used. Our results revealed that after the surgery, most of the participants had a little trouble doing strenuous activities, like carrying a heavy shopping bag or a suitcase (42.9%) or walking long distances (37.3%), while more than half had no trouble walking short distances outside of the house (56.2%) and about one-third (31.7%) had a little trouble in walking short distances. Most respondents did not need to stay in bed or a chair during the day (39%) or help with eating, dressing, washing, or using the toilet (74.9%). Most participants were slightly restricted in their work or other daily activities (42.5%) and slightly restricted in pursuing their hobbies or other leisure time activities (41.3%).

After the surgery, the majority of the respondents did not experience shortness of breath (48.3%), loss of appetite (35.3%), vomiting (46.9%), constipation (43.6%), or diarrhea (53.9%), while about one-third experienced slightly from shortness of breath (34.4%), loss of appetite (34%), vomiting (31.9%), constipation (31.9%), and diarrhea (26.6%). Most of the participants experienced slight pain (37.6%), the need to rest (32.2%), trouble sleeping (37.5%), weakness (38.2%), slight nausea (37.8%), and tiredness (38.8%).

38.6% of the participants reported that their pain did not interfere with their daily activities, and 59.7% had no difficulty concentrating on things like reading a newspaper or watching television. The majority of the respondents did not experience stress (42.3%), worry (42.1%), annoyance (41.9%), or depression (50.2%), while about a one-third experienced slight stress (35.5%), worry (35.7%), annoyance (33.8%), and depression (27.4%). Also, most of the participants did not have difficulty remembering things (56%), and their physical condition or medical treatment did not interfere with their family life (65.4%), social activities (64.5%), or financial status (67.4%) (Table 2).

**Table 2:** Prevalence of post-cholecystectomy syndrome (PCS)

After the surgery:	Not at All	A Little	Quite a Bit	Very much
	n (%)			
1. Do you have any trouble doing strenuous activities, like carrying a heavy shopping bag or a suitcase?	163 (31.5)	222 (42.9)	81 (15.6)	52 (10)
2. Do you have any trouble taking a long walk?	170 (32.8)	193 (37.3)	91 (17.6)	64 (12.4)
3. Do you have any trouble taking a short walk outside of the house?	291 (56.2)	164 (31.7)	38 (7.3)	25 (4.8)
4. Do you need to stay in bed or on a chair during the day?	202 (39)	174 (33.6)	87 (16.8)	55 (10.6)
5. Do you need help with eating, dressing, washing yourself or using the toilet?	388 (74.9)	80 (15.4)	32 (6.2)	18 (3.5)
6. Were you limited in doing either your work or other daily activities?	199 (38.4)	220 (42.5)	68 (13.10	31 (6)
7. Were you limited in pursuing your hobbies or other leisure time activities?	205 (39.6)	214 (41.3)	65 (12.5)	34 (6.6)
8. Were you short of breath?	250 (48.3)	178 (34.4)	54 (10.4)	36 (6.9)
9. Have you had pain?	141 (27.2)	195 (37.6)	110 (21.2)	72 (13.9)
10. Did you need to rest?	116 (22.4)	167 (32.2)	128 (24.7)	107 (20.7)
11. Have you had trouble sleeping?	190 (36.7)	194 (37.5)	77 (14.9)	57 (11)
12. Have you felt weak?	172 (33.2)	198 (38.2)	89 (17.2)	59 (11.4)
13. Have you lacked appetite?	183 (35.3)	176 (34)	84 (16.2)	75 (14.5)
14. Have you felt nauseated?	181 (34.9)	196 (37.8)	73 (14.1)	68 (13.3)
15. Have you vomited?	243 (46.9)	165 (31.9)	58 (11.2)	52 (10)
16. Have you been constipated?	226 (43.6)	165 (31.9)	67 (12.9)	60 (11.6)
17. Have you had diarrhea?	279 (53.9)	138 (26.6)	58 (11.2)	43 (8.3)
18. Were you tired?	142 (27.4)	201 (38.8%)	99 (19.1)	76 (14.7)
19. Did pain interfere with your daily activities?	200 (38.6)	197 (38)	78 (15.1)	43 (8.3)
20. Have you had difficulty concentrating on things like reading a newspaper or watching television?	309 (59.7)	128 (24.7)	50 (9.7)	31 (6)
21. Did you feel tense?	219 (42.3)	184 (35.5)	68 (13.1)	47 (9.1)
22. Did you worry?	218 (42.1)	185 (35.7)	66 (12.7)	49 (9.5)
23. Did you feel irritable?	217 (41.9)	175 (33.8)	71 (13.7)	55 (10.6)
24. Did you feel depressed?	260 (50.2)	142 (27.4)	61 (11.8)	55 (10.6)
25. Have you had difficulty remembering things?	290 (56)	133 (25.7)	60 (11.6)	35 (6.8)
26. Has your physical condition or medical treatment interfered with your family life?	339 (65.4)	109 (21)	43 (8.3)	27 (5.2)
27. Has your physical condition or medical treatment interfered with your social activities?	334 (64.5)	116 (22.4)	42 (8.1)	26 (5)
28. Has your physical condition or medical treatment caused you financial difficulties?	349 (67.4)	104 (20.1)	49 (9.5)	16 (3.1)

**Figure 2:**
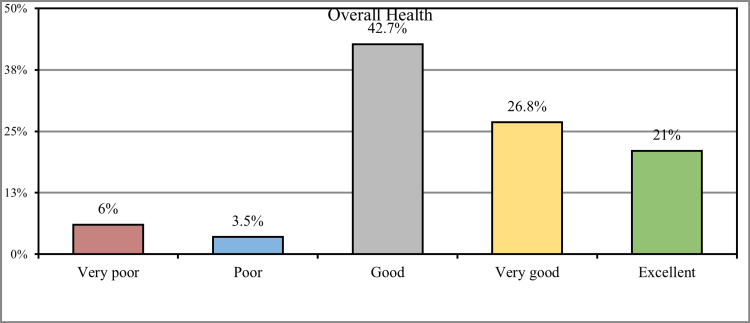
How would you rate your overall health during the past week?

Concerning the overall health status of the participants, 221 (42.7%) stated that they were in good health, 139 (26.8%) had very good health, and 109 (21%) had excellent health, while 31 (6%) of the participants had very poor health and 18 (3.5%) had poor health (Figure 2).

**Figure 3:**
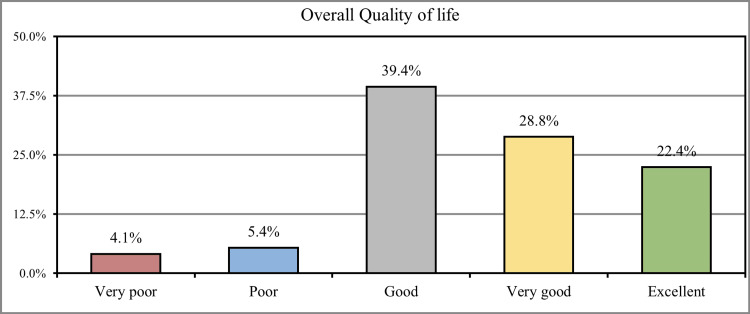
How would you rate your overall quality of life during the past week?

When participants were asked to rate the overall quality of their lives, 221 (39.4%) stated that they had a good quality of life, 139 (28.8%) had a very good quality of life, and 109 (22.4%) had an excellent quality of life. In comparison, 31 (4.1%) of the participants had a very poor quality of life, and 18 (5.4%) had a poor quality of life (Figure 3).

The results revealed that there were no significant associations between age, nationality, and residence with the QLQ-C30 score (P-value = 0.152, 0.262, 0.071, respectively). A significant association was observed between gender and QLQ-C30 score (P-value < 0.001); females had higher scores compared to males (Table 3).

**Table 3:** Association between socio-demographic data and QLQ-C30 score

Variable	Category	QLQ-C30 score		P-value
		Mean	SD	
Age (years)	< 18	55.16	19.95	0.152
	19-25	52.10	16.72	
	26-35	51.42	17.98	
	36-45	51.54	16.53	
	46-55	52.97	17.19	
	> 55	60.59	23.35	
Gender	Male	47.70	15.38	< 0.001
	Female	54.28	18.18	
Nationality	Saudi	52.81	17.61	0.262
	Non-Saudi	49.74	18.67	
Residence	Al-Baha	55.08	17.62	0.071
	Al-Jouf	56.14	8.47	
	Northern borders	43.00	12.59	
	Riyadh	53.19	21.00	
	Eastern Region	48.09	17.29	
	Al-Qassim	42.19	15.12	
	Madinah	56.61	19.49	
	Tabuk	57.55	21.67	
	Jizan	50.48	17.24	
	Hail	48.13	14.69	
	Aseer	47.89	19.15	
	Makkah	53.57	16.29	
	Najran	53.25	12.20	

## Discussion

Determining the prevalence of PCS is important. Even though it is a temporary diagnosis, it could result in many adverse effects on a patient's social life and might affect the quality of life and sense of well-being of most patients post-cholecystectomy [17].

The current study aimed to determine the prevalence of PCS among the general population of Saudi Arabia.

Nearly one-third (29.5%) of the participants were between 19 to 25 years old, and the vast majority were Saudi Arabian (91.1%). More than one-third of the participants (36.9%) were residents of Makkah. The average preoperative weight of the participants was 71 kg. More than one-third of participants had healthy weight preoperatively (36.7%) or currently (37.8%). One-quarter (26.4%) of the participants had a history of chronic diseases. The most reported chronic disease among the study participants was hypertension (37.2%), followed by asthma (36.5%), diabetes mellitus (33.6%), and heart disease (5.1%), while 10.9% had other chronic diseases. This was consistent with the findings reported in the study conducted by Chen et al., which reported the association between cholecystectomy and previous gallstone disease and metabolic syndrome, including DM, hypertension, and cardiovascular diseases [18].

Most of the participants had a little trouble doing strenuous activities, like carrying a heavy shopping bag or a suitcase (42.9%) or walking long distances (37.3%), while more than half (56.2%) had no trouble walking short distances outside of the house. More than one-third (39%) of the respondents did not need to stay in bed or a chair during the day. Less than half of the participants (42.5%) were slightly restricted in their work or other daily activities; also, they were slightly restricted in pursuing their hobbies or other leisure time activities (41.3%). About one-third experienced shortness of breath (34.4%), loss of appetite (34%), vomiting (31.9%), constipation (31.9%), and diarrhea (26.6%). Most of the participants experienced slight pain (37.6%), need to rest (32.2%), trouble sleeping (37.5%), and weakness (38.2%), the percentage of the previously mentioned symptoms were found to be higher compared to the parallel study which carried out by Talsethet al. in which most of the previously mentioned symptoms were reported in only less than 10% of the participants, and this could be attributed to genetic and environmental factors [19].

More than one-third (38.6%) of the participants had pain, but no interference with their daily activities, and more than half (59.7%) had no difficulty concentrating on things like reading a newspaper or watching television. About one-third experienced slight stress (35.5%), worry (35.7%), annoyance (33.8%), and depression (27.4%). This was consistent with the findings of the congruent study conducted by Jen et al., in which post-cholecystectomy pain could result in depression, subsequently affecting the quality of life [20].

Regarding the overall health status of the participants, less than half (42.7%) stated that they had good health, 139 (26.8%) were in very good health, and 109 (21%) had excellent health. In comparison, 31 (6%) of the participants had very poor health, and 18 (3.5%) had poor health; these percentages were found to be higher compared to the other study conducted by Dalboet al. in which only 50% of participants were having a positive perception about their health [21].

More than one-third (39.4%) stated that they had a good quality of life, (28.8%) had a very good quality of life, and (22.4%) had an excellent quality of life, while (4.1%) of the participants had a very poor quality of life and (5.4%) had poor quality of life, similar findings were found in the parallel study conducted by Atif et al. which also revealed that most of the participants had improved quality of life following cholecystectomy [22].

There were no significant associations between age, nationality, and residence with the QLQ-C30 score. A significant association was observed between gender and QLQ-C30 score. Females had higher scores than males, which contradicts another study by Michelson et al. in which women were found to have lower quality-of-life scores compared to males [23].

Due to the self-reported questionnaire used in this study, there are some limitations, including some personal bias due to some participants answering the questions randomly. As well, the questionnaire was distributed online, which may result in some sampling bias toward younger participants who regularly use online means.

## Conclusions

The prevalence of post-cholecystectomy syndrome was found to be high. Participants had good satisfaction with their health. Females reported higher quality-of-life scores than males. These findings should be considered by Saudi healthcare professionals to raise awareness of the symptoms and risks of PCS in the Saudi community.
